# The re-emergence of a data desert for maternal survival is a serious risk amid global health funding cuts

**DOI:** 10.1136/bmjgh-2025-020852

**Published:** 2026-05-21

**Authors:** Ursula Gazeley, Veronique Filippi, Oona M R Campbell, Wendy J Graham

**Affiliations:** 1 Leverhulme Centre for Demographic Science, Nuffield Department of Population Health, University of Oxford, Oxford, UK; 2 Nuffield College, University of Oxford, Oxford, England, UK; 3 Department of Infectious Disease Epidemiology and International Health, Faculty of Epidemiology and Public Health, London School of Hygiene & Tropical Medicine, London, England, UK

**Keywords:** Global Health, Health systems, Maternal health, Pregnancy

**Summary box**

- Severe disruptions to global health financing threaten maternal health progress by destabilising fragile health systems and reversing gains in survival.
- Funding cuts also place maternal health data systems at risk, jeopardising countries’ ability to track maternal mortality, evaluate programmes, and target resources where they are needed most.
- A coordinated international response is needed to secure renewed investment in nationally-led data infrastructure and prevent a growing void in the information required to improve maternal and newborn outcomes.
- Strengthening local and regional ownership is critical to ensuring the long-term sustainability and accountability for these data systems amid shifting global political priorities.

## Main

 Unprecedented disruptions to the global health landscape are underway. President Trump’s 2025 shutdown of the US Agency for International Development (USAID) eliminated an estimated $12–15 billion in annual global health funding,^1^ while the USA’s withdrawal from the World Health Organization (WHO) represents a loss of over 20% of WHO’s total budget.^2^ The US government also terminated $377 million in grants to the United Nations Population Fund (UNFPA).^3^ These cuts were followed by significant reductions in aid budgets across several European countries,^4^ Canada,^5^ and other high income donors, including a 40% reduction in the UK Foreign, Commonwealth and Development Office (FCDO) budget.^6^ Jointly, these developments mark a dismal new era for global health financing.

The speed and scale of foreign assistance funding cuts over the past year present an urgent threat to progress in reducing maternal mortality—an outcome acutely sensitive to funding shocks.^7 8^ Progress achieved since 2000 to improve maternal survival^9^ may be rapidly reversed when financial support is withdrawn from already fragile health systems, further entrenching inequities in reproductive outcomes.

Such an impact of funding cuts on health systems is perhaps obvious. Less well appreciated is the parallel risk to the data infrastructure required to capture trends and identify where, and for whom, the need is greatest. There is a real risk of recreating the data desert in maternal survival that characterised the field over 45 years ago, when the first Safe Motherhood Initiative was launched. As maternal health epidemiologists, we believe this risk threatens the foundation of efforts to improve survival of women and their newborns. A void in data infrastructure will severely limit the effective planning, delivery and evaluation of major programmes, such as antenatal and delivery care, and obscures the needs of the most vulnerable and hardest to reach groups.

The latest global estimates on maternal mortality for 2000–2023 released in 2025 shows that the world is seriously off-target to meet Sustainable Development Goal 3.1: to reduce the global Maternal Mortality Ratio to below 70 by 2030.^9^ However, with cuts already taking hold, these figures may already underestimate the true toll of maternal mortality.

Funding cuts also threaten the future generation of these estimates, creating a substantial gap in evidence as we approach 2030. This risk has been heightened significantly by the termination of USAID and its funding for the Demographic and Health Survey (DHS) Program. For over 40 years, the DHS Program has delivered high-quality population-level estimates of maternal mortality and intervention coverage. In countries without reliable Civil Registration and Vital Statistics (CRVS) systems, DHS data may be the sole input for the UN Joint Agency’s maternal mortality estimates.^9^ The DHS Program also tracks vital maternal and child health coverage metrics, essential to guide government decision-making and resource allocation. The dismantling of USAID disrupted ongoing DHS data collection in 25 countries with immediate effect,^10 11^ highlighting the fragility of core maternal mortality data to the global political economy. Three-year emergency bridge funding from the Gates Foundation in July 2025 provided essential interim funding to ICF International to maintain the DHS Archive and complete some ongoing surveys,^12^ but the long-term sustainability of the DHS Program remains uncertain.^13^ This instability poses serious threats to the continued availability of nationally-representative maternal mortality data across low- and middle-income countries.

The funding crisis may also affect other maternal mortality data infrastructure. Many low- and middle-income countries rely on WHO, UNFPA, and bilateral support to improve CRVS coverage and compliance with International Classification of Diseases (ICD) standards.^14^ Similarly, Health Management Information Systems (HMIS) and Maternal and Perinatal Death Surveillance and Response (MPDSR) systems provide crucial insights into service uptake and quality of care, respectively. The loss of funding and technical support jeopardises the continuity and effectiveness of these vital information sources. As funding evaporates, countries with more limited resources face impossible choices between strengthening data systems and maintaining essential health programmes. This creates a self-perpetuating cycle: the countries that most need information to guide action will be the least equipped to generate it.

Data are powerful and political. As the COVID-19 pandemic demonstrated, undermining data generation can obscure the scale of a crisis and diminish accountability. Without robust maternal health data, the true toll of adverse pregnancy outcomes on women’s lives remains unknown. This weakening of data systems may reflect a broader strategy that reduces accountability and limits external scrutiny.

Immediate, decisive action is required to reduce the risk of recreating a data desert for maternal survival in the 21st century. We call for a global convening—a multistakeholder process led by United Nations agencies, their regional offices, and other multilateral stakeholders—to support countries in assessing options and charting a proactive way forward in response to current funding challenges. This should involve strengthening nationally-managed data systems that are more resilient to domestic and global political shifts. National governments must also maintain domestic support for population-level maternal mortality surveillance systems. A coordinated response is urgently needed to prevent widening data gaps that threaten fragile progress in maternal and newborn survival.

## Data Availability

There is no data in this work.
